# Chloracne and Hyperpigmentation Caused by Exposure to Hazardous Aryl Hydrocarbon Receptor Ligands

**DOI:** 10.3390/ijerph16234864

**Published:** 2019-12-03

**Authors:** Masutaka Furue, Gaku Tsuji

**Affiliations:** 1 Department of Dermatology, Graduate School of Medical Sciences, Kyushu University, Maidashi 3-1-1, Higashiku, Fukuoka 812-8582, Japan; gakku@dermatol.med.kyushu-u.ac.jp; 2 Research and Clinical Center for Yusho and Dioxin, Kyushu University, Maidashi 3-1-1, Higashiku, Fukuoka 812-8582, Japan; 3 Division of Skin Surface Sensing, Graduate School of Medical Sciences, Kyushu University, Maidashi 3-1-1, Higashiku, Fukuoka 812-8582, Japan

**Keywords:** chloracne, hyperpigmentation, dioxin, aryl hydrocarbon receptor, reactive oxygen species, epidermal terminal differentiation, melanocytes

## Abstract

Dioxins and dioxin-like compounds are environmental pollutants that are hazardous to human skin. They can be present in contaminated soil, water, and air particles (such as ambient PM_2.5_). Exposure to a high concentration of dioxins induces chloracne and hyperpigmentation. These chemicals exert their toxic effects by activating the aryl hydrocarbon receptor (AHR) which is abundantly expressed in skin cells, such as keratinocytes, sebocytes, and melanocytes. Ligation of AHR by dioxins induces exaggerated acceleration of epidermal terminal differentiation (keratinization) and converts sebocytes toward keratinocyte differentiation, which results in chloracne formation. AHR activation potently upregulates melanogenesis in melanocytes by upregulating the expression of melanogenic enzymes, which results in hyperpigmentation. Because AHR-mediated oxidative stress contributes to these hazardous effects, antioxidative agents may be potentially therapeutic for chloracne and hyperpigmentation.

## 1. Introduction

Health problems induced by environmental pollutants are an important issue. Environmental polycyclic and halogenated aromatic hydrocarbons, such as 2,3,7,8-tetrachlorodibenzo-*p*-dioxin (TCDD), polychlorinated dibenzo-p-dioxins (PCDDs), polychlorinated dibenzofurans (PCDFs), polychlorinated biphenyls (PCBs), and benzo[*a*]pyrene (BaP) are high-affinity ligands for aryl hydrocarbon receptors (AHRs), namely, dioxin receptor [1,2,3,4,5]. To sense these chemicals, AHR is abundantly expressed in skin cells, including epidermal keratinocytes [1,2,3,4,5]. Therefore, skin is one of the most important target organs for these environmental AHR ligands. 

The toxic potency of these dioxins and dioxin-like compounds are variable in humans and other mammals. To estimate the total body burden, the toxic equivalency factor (TEF) has been defined for each compound by the World Health Organization (WHO) [6]. The body burden of these molecules is calculated by the sum of toxic equivalency (TEQ) of each compound (TEF × concentration of the compound) [6,7]. Exposure to high TEQ concentration of dioxins manifests various acute systemic signs and symptoms, including general malaise, cough/sputum, diarrhea, headache, nausea, arthralgia, and pain/dysesthesia of extremities [8,9,10,11]. In addition, the most prominent clinical findings are chloracne and hyperpigmentation [9,10,11,12]. Similar skin disorders are induced by other endocrine-disrupting chemicals [13]. 

In Japan, chloracne and hyperpigmentation are present in Yusho, which occurred in Japan in 1968 by mass food poisoning with high concentrations of PCDFs and related compounds [12,14,15]. Because these compounds are extremely lipophilic and structurally stable, high concentrations of PCDF are still detectable in the blood of those exposed, even 50 years after the outbreak [16,17,18]. Chloracne has been typical of other incidents of dioxin poisoning; examples include TCDD exposure from an industrial accident in Seveso, Italy [9]; the Yucheng illness, a mass poisoning in Taiwan caused by PCDF [10]; and the poisoning of former Ukrainian President Victor Yushchenko with TCDD [11]. Hyperpigmentation was noted in Asian individuals with darker skin in the Yusho (Japan) and Yucheng (Taiwan) incidents, but was also recognized in President Yushchenko [9,10,11,12]. Air pollutants, including ambient particulate matter of up to 2.5 µm in diameter (PM_2.5_), contain high concentrations of polycyclic aromatic hydrocarbons and BaP [19]. Notably, facial hyperpigmentation is significantly associated with exposure to PM_2.5_ in Chinese women [20]. In this article, we will review the current evidence on chloracne and hyperpigmentation induced by AHR activation.

## 2. AHR Signals and Oxidative Stress in Epidermal Keratinocytes

AHR is a ligand-activated transcription factor [21]. In the absence of ligands, AHR resides in the cytoplasm, where it forms a protein complex with heat shock protein 90 (HSP90), hepatitis B virus X-associated protein 2 (XAP-2), and p23 [22,23]. After ligand binding, AHR dissociates from the cytoplasmic complex, and a nuclear translocation site of AHR is exposed. Then, AHR is translocated into the nucleus, where it dimerizes with AHR-nuclear translocator (ARNT), binds DNA-responsive elements called xenobiotic responsive elements (XRE), and upregulates the transcription of target genes, such as phase I metabolizing enzyme cytochrome P450 (CYP) members (i.e., *CYP1A1*, *CYP1A2*, and *CYP1B1*) [1,2,3,4,5,21,24,25]. 

Environmental dioxins such as TCDD activate AHR and upregulate CYP1A1, CYP1A2, and CYP1B1 expression [1,26,27]. Human keratinocytes abundantly express CYP1A1 and, to a lesser extent, CYP1B1, but not CYP1A2 [28]. As TCDD is structurally stable, the induction of TCDD-AHR-mediated CYP1A1 expression may be sustained for a long period [26,29]. The metabolizing process of CYP1A1 generates excessive amounts of reactive oxygen species (ROSs) and induces oxidative damage in the cell [1,26,27,30]. As proof of this, TCDD-induced ROS production was cancelled in AHR-silenced or CYP1A1-silenced human aortic endothelial cells [26]. Because CYP1B1 silencing did not affect TCDD-induced ROS generation, the AHR/CYP1A1 axis is likely to be crucial for generating cellular oxidative stress by environmental dioxins [26]. In mice, a chemical carcinogen, β-naphthoflavone, also activates CYP1A1 and CYP1A2 via AHR activation [31]. β-naphthoflavone induces mitochondrial ROS generation; however, this is attenuated by the AHR inhibitor or *Cyp1a1/1a2*-silencing in mice [31]. CYP1A1-mediated oxidative stress is responsible, at least in part, for the production of proinflammatory cytokines such as interleukin (IL) 1, IL-6, and IL-8 in human keratinocytes [32,33]. AHR activation also induces the production of proinflammatory cytokines in sebocytes [34,35].

To counteract the oxidative stress, antioxidative mechanisms operate simultaneously after AHR activation. Ligation of AHR also activates the antioxidative transcription factor nuclear factor erythroid 2–related factor 2 (NRF2) and upregulates the expression of phase II antioxidative enzymes, such as glutathione *S*-transferases, heme oxygenase 1, nicotinamide adenine dinucleotide phosphate (NADPH) dehydrogenase, quinone 1, glutathione *S*-transferases, and uridine 5′-diphospho-glucuronosyltransferases transferases [24,36,37,38,39,40,41,42]. Dioxins activate the AHR/NRF2 battery [42,43,44]; however, their powerful AHR-mediated CYP1A1 expression may induce far more oxidative stress, such that it cannot be extinguished by the AHR/NRF2 antioxidative system. ROS-mediated oxidative stress induces DNA damage and upregulates the production of inflammatory cytokines and chemokines in keratinocytes [27,33,45]. 

In addition to CYP1A1 and ROS upregulation, AHR exerts a variety of mutually-interacting signal transduction. TCDD upregulates phosphorylation of epidermal growth factor receptor (EGFR), ERK, and p38 MAPK, then augments the proliferation and epithelial-mesenchymal transition of human palatal epithelial cells in an AHR-dependent manner [46]. BaP promotes gastric carcinoma cell proliferation by c-MYC activation via the AHR-ERK pathway [47]. On the other hand, EGFR signaling inhibits the AHR-mediated CYP1A1 induction, because EGFR and AHR competitively share a common coactivator p300 for their transcriptional activity in keratinocytes [48]. The transcription and translation of AHR and ARNT is regulated by c-MYC, and AHR-ARNT is partly involved in c-MYC-mediated protein expression [49]. These studies stress the multifaceted and occasionally conflicting role of AHR in the proliferation and differentiation of epithelial cells.

## 3. AHR Signaling Accelerates Epidermal Terminal Differentiation

The mammalian epidermis is composed of stratified squamous keratinocytes that protect the body against injuries caused by external and environmental chemicals. Epidermal keratinocytes divide in the basal layer and move up into the spinous, granular, and outermost cornified layer, which plays an essential role in skin barrier formation [50]. This maturation process is accomplished by sequential and coordinated cross-linking by transglutaminase-1 and -3 of ceramides and various epidermal differentiation complex (EDC) proteins, such as involucrin (IVL), loricrin (LOR), and filaggrin (FLG) [50]. Mounting evidence indicates that the AHR signal plays a crucial role in epidermal terminal differentiation [3,4,51]. In parallel, both *Ahr*-deficient and *Ahr*-transgenic mice show an abnormality in keratinization [52,53], and a severe abnormality in keratinization is also observed in *Arnt*-deficient mice [54,55].

In utero exposure to TCDD accelerates the expression of FLG and LOR, together with earlier maturation of the epidermal permeability barrier in fetal mouse skin [56,57]. In a three-dimensional skin-equivalent model, TCDD accelerates the differentiation of human keratinocytes [58]. The expression of FLG is detected only in the granular layer in a vehicle-treated skin equivalent, whereas it is markedly enhanced and even detected in the keratinocytes of the spinous layer in TCDD-treated samples [58]. The expression of IVL is found only in the suprabasal keratinocytes in vehicle-treated skin equivalents; however, TCDD accelerates its expression in basal keratinocytes [58].

The upregulated expression of EDC proteins and accelerated terminal differentiation is also evident in monolayer keratinocyte culture by AHR activation [48,59,60,61,62]. Kennedy et al. [59] have shown that TCDD upregulates the expression of 40% of the EDC genes and 75% of the genes required for de novo ceramide biosynthesis without affecting the levels of cholesterol and free fatty acids. The AHR-mediated upregulation of EDC proteins is cancelled in AHR-deficient keratinocytes or by AHR antagonists [62]. Moreover, the accelerated epidermal differentiation by TCDD is blocked in the presence of antioxidant agents, indicating the critical role of ROSs generated by AHR activation with TCDD [59]. 

In physiological conditions, AHR is continuously activated by endogenous and exogenous AHR ligands [3,51,63,64]. Ultraviolet radiation induces a photodimerization of endogenous tryptophan and generates 6-formylindolo[3,2-*b*]carbazole (FICZ) [65]. Cutaneous commensal microbiota metabolize tryptophan to indole-3-aldehyde [64]. Intestinal microbiota are a good source of AHR ligands, such as indirubin [66,67]. Both FICZ and indirubin are high-affinity endogenous AHR ligands that could feasibly upregulate the expression of EDC proteins, such as FLG and IVL [63,66,68,69,70]. Therefore, either physiological or environmental AHR ligands accelerate epidermal differentiation. The physiological and endogenous ligands are rapidly degraded by the AHR-induced CYP1A1 [65] so that their AHR-activating ability may be transient. Although the mechanism leading to chloracne by dioxins is not fully understood, structurally-stable dioxins may induce exaggerated and sustained acceleration of epidermal differentiation. 

## 4. Chloracne Caused by Environmental AHR Ligands 

**Figure 1:**
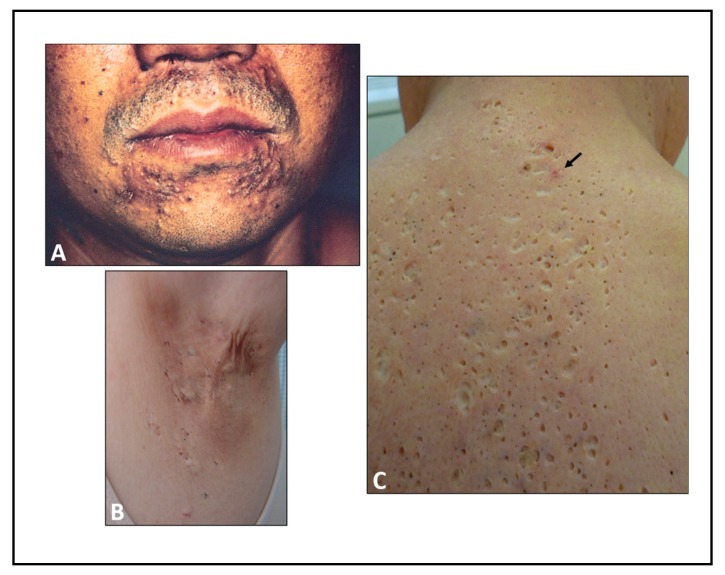
Clinical features of chloracne in patients with Yusho disease, an outbreak that occurred in Japan in 1968. (**A**) Chloracne in the perioral area in 1968. (**B**) Scars and cysts from chloracne in the axilla in 2008. (**C**) Severe crateriform, or punched-out scars, from chloracne on the back in 2008. Inflammatory acneiform eruption still appears frequently (arrow).

Chloracne is characterized by an acne-like eruption with comedones, cysts, and pustules that occurs after exposure to high concentrations of environmental AHR ligands, such as TCDD and PCDF [9,10,11,12,14] (Figure 1; see also [71]). In the Seveso explosion accident, chloracne was also found in children exposed to contaminated air containing high concentrations of TCDD [72]. 

The severity of chloracne is significantly correlated with the blood levels of PCDF in Yusho patients [12]. The histopathology of chloracne, which was well-described by Suskind in 1985 [73], includes hyperkeratinization of the interfollicular epidermis, hyperproliferation and hyperkeratinization of hair follicle cells, gradual loss of sebocytes with shrinkage of sebaceous glands, and infundibular dilatation, eventually leading to comedo or cyst formation [73,74,75].

**Figure 2:**
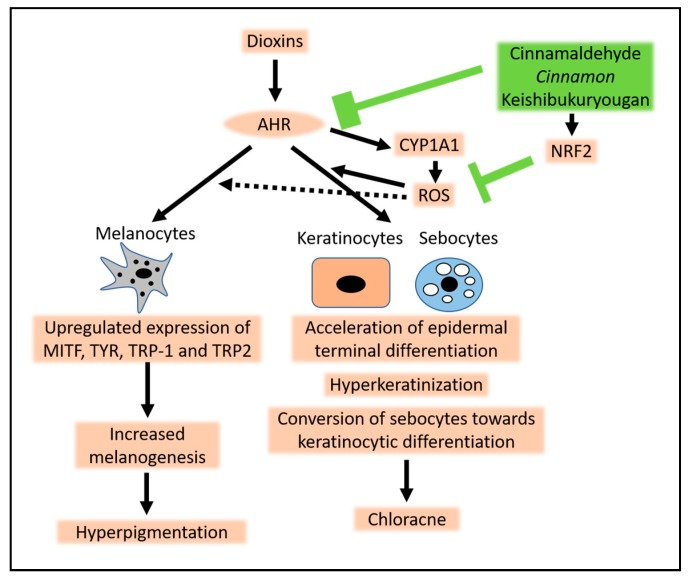
A schema of the pathogenesis of chloracne and hyperpigmentation by dioxins. Dioxins bind to the aryl hydrocarbon receptor (AHR) and induce production of xenobiotic metabolizing enzyme cytochrome p450 1A1 (CYP1A1). CYP1A1 tries to degrade the dioxins, but has little success because dioxins are structurally stable. These unsuccessful efforts give rise to enormous production of reactive oxygen species (ROSs). AHR signaling, together with the oxidative stress, accelerate the epidermal terminal differentiation (i.e., keratinization) in keratinocytes. This also converts sebocytes toward keratinocytic differentiation, which results in the development of chloracne. AHR activation by dioxins also upregulates the expression of melanogenic genes, including microphthalmia-associated transcription factor (MITF), tyrosinase (TYR), and tyrosinase-related proteins 1 and 2 (TYRP1 and TYRP2), and increases melanogenesis in melanocytes, resulting in hyperpigmentation. It is not clear whether ROSs are involved in dioxin-induced hyperpigmentation. Cinnamaldehyde, cinnamon, and the cinnamon-containing herbal drug Keishi-bukuryo-gan inhibit the AHR-mediated CYP1A1 expression. They also activate nuclear factor erythroid 2-related factor 2 (NRF2), upregulate gene expression of antioxidative enzymes, neutralize ROSs, and inhibit chloracne formation.

The pathomechanism of chloracne is not fully understood, but current experimental results indicate that it is closely associated with exaggerated acceleration of terminal differentiation of keratinocytes, especially sebocytes (Figure 2). Sebocytes are specialized keratinocytes that produce sebum lipids and constitute sebaceous glands, which are connected to hair follicles [74], and they express high amounts of AHR [74,76,77]. Ligation of AHR by dioxins causes sebocytes to lose their specific features for sebaceous differentiation, including lipogenesis, keratin 7 expression, and epithelial membrane antigen expression [34,74,76,77]. Instead, AHR activation converts sebocytes toward keratinocyte differentiation, upregulating keratin 10 and peroxisome proliferator-activated receptor-δ [74]. Consistent with in vitro sebocyte culture results, ex vivo sebaceous gland cultures show that dioxin induces the shrinkage and disappearance of sebaceous glands [74]. In addition, topical application of TCDD induces epidermal hyperplasia, hyperkeratosis, and sebaceous gland metaplasia toward keratinocyte differentiation in hairless mice [78]. These in vitro, ex vivo, and in vivo results coincide well with the already mentioned histopathological loss of sebocytes and shrinkage of sebaceous glands in chloracne in humans [73,75]. Taken together, dioxin–AHR signaling induces exaggerated acceleration of terminal differentiation in keratinocytes, which results in hyperkeratinization of keratinocytes and conversion of sebocytes to keratinocytes. 

In addition to the acceleration of keratinization, an immunohistological study revealed an activation of EGFR in chloracne [79]. AMP-activated protein kinase (AMPK) is activated by AHR and downregulates protein turnover of the mature sterol regulatory element-binding protein (mSREBP-1), leading to a decrease in the size of sebaceous glands and the number of sebocytes within each gland in the skin [80]. TCDD may affect the stem cells in sebaceous glands [81]. AHR activation stimulates keratinocytes and sebocytes to produce proinflammatory cytokines, such as IL-1α, IL-1β, IL-6, and IL-8, which play an additional role in the development of chloracne [32,33,77]. AHR-mediated cytokine production is dependent on ROS generation [32,33]. Because the AHR-mediated acceleration of keratinocyte differentiation is also dependent on oxidative stress [59], antioxidants may be efficacious for chloracne. We have found that cinnamon (20 μg/mL) and its major constituent, cinnamaldehyde (25 μM), are potent antioxidants and have dual activities: suppression of AHR-induced CYP1A1 expression and activation of the NRF2 antioxidative system [82]. Keishi-bukuryo-gan is a cinnamon-containing herbal drug and 100 μg/mL of Keishi-bukuryo-gan showed the similar level of inhibitory action on the AHR-induced CYP1A1 expression, as did 20 μg/mL of cinnamon in vitro [82]. In a clinical setting, three months of oral administration of Keishi-bukuryo-gan (3.75 g/day, bis in die) improved general fatigue, chloracne, and cough/sputum in Yusho patients [15]. Keishi-bukuryo-gan also improved their quality of life as assessed by the self-reported questionnaire SF-36 [15]. Although the in vivo dose of cinnamon is much less than its in vitro effective dose, the daily intake of cinnamon may be beneficial for those exposed to high levels of dioxins.

## 5. Hyperpigmentation Caused by Environmental AHR Ligands 

**Figure 3:**
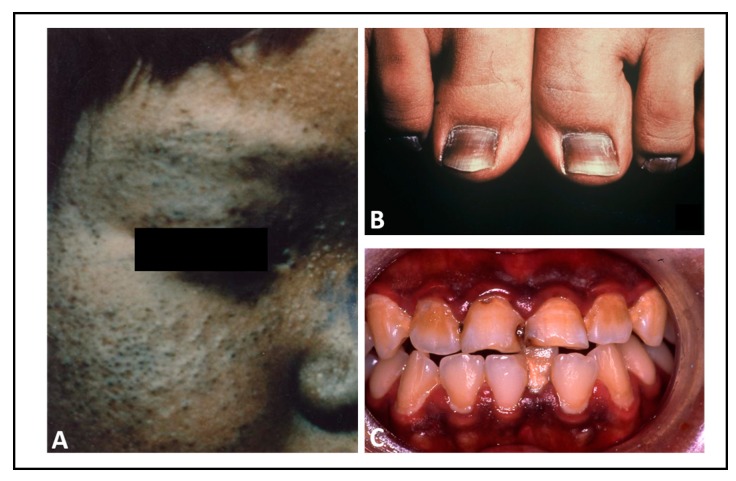
Clinical features of hyperpigmentation in patients with Yusho disease. (**A**) Hyperpigmentation and chloracne on the face. (**B**) Ungual hyperpigmentation. (**C**) Gingival hyperpigmentation.

Cutaneous hyperpigmentation was one of the diagnostic hallmarks in the Yusho and Yucheng diseases [10,14] (Figure 3; see also [71]). PM_2.5_ contains various amounts of dioxin-related compounds and could feasibly activate the AHR signal [19,77]. It has been reported that facial hyperpigmentation is high in people living in air-polluted areas with high PM_2.5_ concentrations in China [20]. Although hyperpigmentation is not functionally problematic, it causes significant cosmetic and psychological distress. In melanocytes, melanin granules are produced by sequential enzymatic reactions by tyrosinase (TYR) and tyrosinase-related proteins 1 and 2 (TYRP1 and TYRP2). The expression of these melanogenic enzymes is upregulated by the microphthalmia-associated transcription factor (MITF), which is a key transcriptional regulator in melanogenesis [83,84]. Human and murine melanocytes express functional AHR [85,86,87,88].

Tobacco smoke contains environmental AHR ligands, such as BaP [33,89]. AHR activation by tobacco smoke activates MITF and upregulates the expression of TYR, leading to increased melanogenesis [88,89] (Figure 2). Luecke et al. [87] have also reported that TCDD enhances the expression of TYR and TYRP2 in an AHR-dependent manner and induces the production of melanin. In addition, Abbas et al. [86] showed that the oxidative AHR ligand benzanthrone upregulates TYR activity and increases melanin production in murine melanocytes in vitro [86]. Moreover, topical application of benzanthrone or TCDD induces cutaneous hyperpigmentation and increases histological melanin deposits, together with upregulated protein expression levels of MITF, TYR, TRP1, and TRP2 [86]. These in vitro and in vivo studies support the notion that AHR signaling directly augments melanogenesis and induces hyperpigmentation. However, the involvement of ROSs in melanogenesis is controversial [90,91]. ROS production may not be a prerequisite factor for AHR-mediated hyperpigmentation. 

## 6. Conclusions

Skin is exposed to numerous environmental pollutants. Some of these, such as halogenated aromatic hydrocarbons, including dioxins, are hazardous and induce chloracne and hyperpigmentation in high-concentration exposure. These environmental chemicals strongly activate AHR, which is abundantly expressed in keratinocytes, sebocytes, and melanocytes. Accumulating evidence indicates that AHR ligation by dioxins accelerates epidermal terminal differentiation (keratinization) of keratinocytes and converts sebocytes to a keratinocytic phenotype, leading to chloracne formation. Dioxins also increase the melanogenesis of melanocytes via AHR activation, leading to clinical hyperpigmentation.
